# Experimental study on basic engineering properties of loess improved by burnt rock

**DOI:** 10.1038/s41598-023-38083-z

**Published:** 2023-07-07

**Authors:** Kai Chen, Dan Shao, Zhiqi Liu, Lifeng Chen, Genyi He

**Affiliations:** 1 School of Geology and Mining Engineering, Xinjiang University, Urumqi, 830017 China; 2 School of Resource and Earth Science, China University of Mining & Technology, Xuzhou, 221116 China; 3 State Key Laboratory for Geomechanics and Deep Underground Engineering, Xuzhou, 221116 China; 4 Northwest Bureau of China Metallurgical Geology Bureau, Xi’an, 710000 China; 5 The Second Hydrology Engineering Geology Brigade of Xinjiang Bureau of Geology and Mineral Re-sources, Xinjiang, 831100 Changji China

**Keywords:** Natural hazards, Solid Earth sciences

## Abstract

Modifying the loess foundation effectively solved the deformation and settlement of the building foundation and improved its stability. However, burnt rock-solid waste was often used as filling material and light aggregate, while there were few studies on the engineering mechanical properties of modified soil. This paper proposed a method of burnt rock solid waste-modified loess. Therefore, we conducted compression-consolidation and direct shear tests on burnt rock solid waste-modified loess under different burnt rock contents to explore its improved loess’s deformation and strength characteristics. Then, we used an SEM to investigate the modified loess’s micro-structures under different burnt rock contents. The results showed that as the burnt rock-solid waste particle content continued to increase, the void ratio and compressibility coefficient of the samples with different ranges of burnt rock-solid waste particles gradually decreased with rising vertical pressure, while the compressive modulus increased first, then reduced and then increased with the increase of vertical pressure; the shear strength indexes all showed an increasing trend with the increased content of burnt rock-solid waste particles; when the content of burnt rock-solid waste particles was 50%, the compressibility of mixed soil was the lowest, the shear strength was the largest, and the compaction effect and shear resistance were the best. However, when the content of burnt rock particles was 10–20%, the shear strength of the soil improved significantly within the content range. The mechanism of burnt rock-solid waste to enhance the strength of the loess structure was mainly to reduce the porosity and average area of soil, significantly improve the strength and stability of mixed soil particles, and thus significantly improve the mechanical properties of soil. The results of this research will provide technical support for safe engineering construction and geological disaster prevention and control in loess areas.

## Introduction

Loess is the most widely distributed in Asia. In China, it is mainly distributed in Northwest, North China, and Northeast China, with an area of 62.4 × 10^4^ km^2^^1,2^. Compared with sandy soil and clayey soil, the engineering characteristics of loess are uniquely different. In the pressure range where its structural strength was not damaged or softened, it showed properties such as low compressibility and high strength. However, once a structure becomes damaged, its mechanical performance exhibits characteristics such as yield, softening, and collapsibility, which cause the structure to settle substantially, crack, and tilt, seriously affecting its safety and use^3,4^ Therefore, many safety hazards need consideration as significant challenges to the construction of loess areas. Therefore, exploring a novel, efficient, economical, and environmentally friendly method of loess improvement was necessary and prudent.

Burnt rock is a special kind formed by coal seam spontaneous combustion and baking surrounding rock, widely distributed worldwide^5^. Although coal seam spontaneous combustion caused severe damage to the ecological environment, its combustion products also provided a new type of building material for engineering construction^6^. Liu et al. studied the burnt products from the burning area as a burnt rock deposit. They believed this deposit was ideal for refractories, ceramics, and building materials^7^. Wang et al. believed that the Qianshuihe burnt rock deposit in Xinjiang couldn’t be widely used in cement, refractory, and other building materials. Instead, they initially developed some burnt rock cement composite products^8^. Yang et al. found that burnt rock could also be exploited as a natural light aggregate through lab tests^9^. Zhai et al. applied the new burnt rock-solid waste-filling technology to the coalfield fire in the Gulaben mining area. They achieved outstanding results in equipment development and field use^10^. Liu et al. developed burnt rock-solid waste as a new antifire grouting material to replace loess grout for fire extinguishing operations^11^. Additionally, a uniaxial compression test^12^, shear test^13–15^, and acoustic emission tests^16–18^ investigated burnt rock’s physical and mechanical performance waste. The above studies on burnt rock mainly focus on filling materials, petrological characteristics, and lightweight aggregate. In contrast, research results on burnt rock-solid waste as a modified material for foundation treatment were relatively rare.

Modifying the loess foundation effectively solved the deformation and settlement of the building foundation and improved its stability^19^. Compaction modification technologies such as dynamic and compaction pile methods were widely used in loess areas, have a relatively mature system, and formed a series of technical specifications and standards^20^. Meanwhile, in recent years, many experts and scholars at home and abroad have used physical or chemical modification methods to study the engineering characteristics of adverse foundation soil^21^. Bai et al.^22^ used a collapsibility test, gray relational analysis, and unconfined compressive strength test to study calcium lignosulfonate-modified loess’s collapsibility and mechanical properties. Luo et al.^23^ conducted direct shear tests and unconfined compression tests by adding cement, polypropylene fibers, and loess samples of SCA-2 soil stabilizer composites and found that the compressive strength indexes of modified loess had a significant impact on foundation settlement. Cheng et al.^24^ proposed using microorganism-induced calcium carbonate deposition technology to modify loess mechanical performance. The calcium carbonate cemented soil particles induced by microorganisms significantly improved the connection strength between soil particles, thereby considerably improving the mechanical properties of soil. Zhang^25^ used a variety of used tire rubber particles as additives and cement as a modifier. Through laboratory tests and theoretical analysis, he studied the engineering properties and modification mechanism of used tire rubber-modified soil. Zhong et al.^26^ prepared modified loess samples with different mix ratios by adding fly ash static pressure to loess, carried out a dynamic triaxial test after different dry–wet cycles, and proposed the optimal mix ratio of fly ash-modified loess. Han Y et al.^27,28^ used the solid nano-calcium carbonate (nano-CaCO_3_) derived from gaseous carbon dioxide is used as an admixture to UHPC, which indirectly realizes the capture and storage of carbon dioxide by UHPC.

The research on modification methods of adverse foundation mainly focused on used tire particles, lignin, nano-graphite powder, and fly ash. At the same time, there needed to be more research on burnt rock-solid waste, unique to mining areas in western China, as an additive. Meanwhile, from the perspective of the environmental protection economy, burnt rock as a secondary utilization resource can effectively reduce the cost of building foundation treatment and exploit local waste to achieve the effect of environmental protection. Furthermore, Urumqi was the core city of the “Silk Road Economic Belt” and occupied an essential position in the “Belt and Road” initiative^29^^.^ In this study, burnt rock was employed to modify loess to improve the mechanical performance of loess and provided new technical ideas for the engineering construction of loess areas. The deformation and strength parameters of loess samples were analyzed by compression-consolidation test and shear strength test while controlling different blending amounts of loess and burnt rock-solid waste particles to reveal the microcosmic mechanism of burnt rock-solid waste particles-modified loess engineering properties through SEM, laying a theoretical foundation for the future application of this technology for loess areas.

## Materials and methods

### Materials

The loess used in this research was taken from a construction site in Urumqi, China. The soil layer was widely distributed in the site, with a 1.4–9.4 m thickness and an average of 4.75 m. Tables 1, 2 and 3 show some of its fundamental physical, mechanical, and chemical property data. As observed, the collapsibility coefficients of the loess strata were all greater than 0.015, which belonged to the self-weight collapsibility loess and the collapsibility grade in class II (medium). The burnt rock sample used was the burnt rock produced by the Urumqi Xishan coalfield fire, and the specific sampling location was near the Baituyao coal mine in Urumqi, China.

**Table 1:** Physical properties of loess in study area.

The number of the borehole	Sample depth m	Water content *ω* %	Density *ρ* g/cm^3^	Dry density *ρ*_*d*_ g/cm^3^	Soil particle proportion *Gs*	Void ratio *e*	Degree of saturation *Sr* %	Porosity *n* %	Liquid limit *ω*_*L*_ %	Plastic limit *ω*_*P*_ %	Plasticity index *I*_*P*_	Liquidity index *I*_*L*_
TK22-1	2.3–2.5	13.5	1.56	1.37	2.70	0.964	37.8	49.1	21.5	14.6	6.9	− 0.2
TK22-2	3.3–3.5	11.4	1.72	1.54	2.70	0.748	41.0	42.8	21.3	14.5	6.8	− 0.5
TK22-3	4.3–4.5	6.0	1.65	1.56	2.70	0.735	22.0	42.4	20.6	14.0	6.6	− 1.2

**Table 2:** Mechanical properties of loess in the study area.

The number of the borehole	Sample depth m	Compression factor *a*_v0.1~0.2_ MPa^–1^	Compression modulus *E*_s0.1~0.2_ MPa	Coefficient of collapsibility *δs*	Coefficient of self-weight collapsibility *δ*_*ZS*_	Initial collapse pressure kPa
TK22-1	2.3–2.5	0.30	6.55	0.112	0.025	–
TK22-2	3.3–3.5	0.16	10.93	0.038	0.012	80
TK22-3	4.5–4.5	0.18	9.64	0.141	0.057	–

**Table 3:** Chemical properties of loess in the study area.

The number of the borehole	Sample depth m	HCO_3_ mg/kg	Cl^–^ mg/kg	SO_4_^2–^ mg/kg	Ca^2+^ mg/kg	Mg^2+^ mg/kg	Na^+^ + K^+^ mg/kg	PH value	Total soluble salt mg/kg	Molar ratio Cl^–^/2SO_4_^2–^	Sodium sulfate content %
TK11-1	0.8–1.0	331	560	703	240	29	494	7.6	2360	1.08	0.04
TK11-2	1.8–2.0	265	415	818	72	29	623	7.5	2220	0.69	0.11
TK11-3	2.8–3.0	331	456	559	72	15	578	7.5	2010	1.11	0.09
TK11-4	3.8–4.0	463	539	530	144	15	585	7.6	2280	1.38	0.07
TK11-5	4.8–5.0	463	539	732	96	15	737	7.6	2580	1.00	0.12
TK11-6	5.8–6.0	397	477	732	90	15	672	7.5	2390	0.88	0.11

### Sample preparation

**Figure 1:**
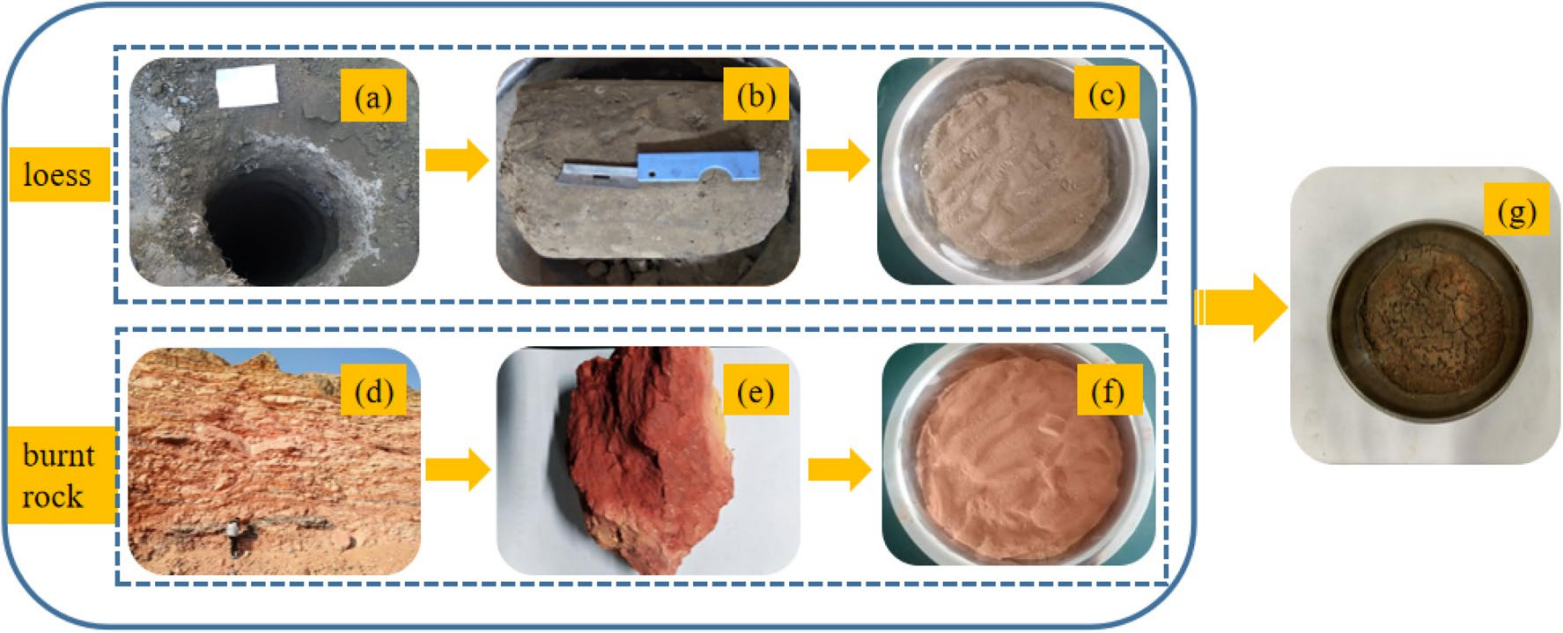
Test sample preparation process. (**a**) Field sampling exploration well; (**b**) Undisturbed loess poplar; (**c**) Loess particles; (**d**) Sampling location for burnt rock in the field; (**e**) Burnt rock block; (**f**) Burnt rock particles; (**g**) Mixed samples of burnt rock and loess.

The collected loess and burnt rock samples were crushed with a multi-functional pulverizer and retained with a particle size of less than 0.075 mm, and the burnt rock particles with a particle size of less than 0.5 mm by sieving. Then, the burnt rock particles and loess were mixed according to the contents of 0%, 10%, 20%, 30%, 40%, and 50% and sprayed with the estimated amount of water with a watering can to mix well. The prepared mixed soil sample was also put into a fresh-keeping bag, tightly tied, and stored in an airtight container for 24 h. The preparation process of the samples is shown in Fig. 1a–g.

In this paper, content is defined as the percentage of the mass of burnt rock particles to the mass of mixed soil in a dry state:

1 $$u\,\, \%=\frac{{m}_{a}}{{m}_{s}}\times 100\%,$$

where u is the content, m_a_ is the mass of burnt rock particles, g; m_s_ is the mass of mixed soil, g.

### Methods

#### Compression-consolidation test

This research mainly measured the void ratio e, coefficient of compressibility a, and compressive modulus *E*s of different mixed soil samples and studied its compression characteristics law. And the one-dimensional compression-consolidation test was mainly carried out concerning the standard of geotechnical test methods (GB/T 50123-2019). The applied load was 50 kPa, 100 kPa, 200 kPa, 300 kPa, and 400 kPa, and the standard of sample deformation stability under each load level was that the deformation rate was less than 0.001 mm/h.

#### Shear strength test

This test mainly measured the cohesion c and angle of internal friction ψ of different mixed soil samples and studied the change law of its shear strength indexes for further contents. A strain-controlled direct shear instrument (ZJ) was employed to push four samples with the exact content of burnt rock-solid waste particles under different vertical pressures at an equal shear rate to generate displacement and measure the corresponding shear stress. And the vertical pressures applied were 100 kPa, 200 kPa, 300 kPa, and 400 kPa, respectively. They adopted the standard sample shear rate under different vertical pressures: a displacement rate of less than 0.8 mm/min.

#### Characterization by SEM

FESEM (SU8000) was employed to analyze the microstructure of different mixed soil samples. First, each sample was put in an oven, dried at 107 °C for 24 h, and then crushed the dried sample. Afterward, the sample was spread evenly on the conductive tape with a length and width of about 3 mm, followed by pasting it on the SEM observation platform for SEM observation. Magnifications of 5000 times, 20,000 times, 50,000 times, and 100,000 times were used for compliance, respectively.

This section may be divided by subheadings. It should provide a concise and precise description of the experimental results, their interpretation, as well as the experimental conclusions that can be drawn.

## Results

### Initial physical parameters

The initial physical parameters of the loess and burnt rock particle mixed soil sample are shown in Table 4.

**Table 4:** Initial physical parameter values of samples.

Mixing ratio of burnt rock solid particles *u*/%	Initial water content *ω*_0_/%	Initial amplitude density *ρ*_*0*_/(g·cm^-3^)	Average volumetric weight *G*_*st*_	Initial void ratio *e*_*0*_
0	20%	1.876	26.85	0.7175
10%	20%	1.96	27.1	0.6592
20%	20%	2.043	27.29	0.6029
30%	20%	2.127	27.46	0.5492
40%	20%	2.244	27.62	0.4770
50%	20%	2.345	27.75	0.4200

### Deformation parameters of modified loess: compression-consolidation test

See Table 5 for the void ratio, compression coefficient, and compression modulus data of loess and burnt rock particle mixed soil samples.

**Table 5:** Compression index under different dosage.

Parameter	Vertical pressure *p*/kPa	0%	10%	20%	30%	40%	50%
Void ratio *e*_*i*_	50	0.7007	0.6463	0.5921	0.5399	0.4690	0.4131
	100	0.6887	0.6364	0.5842	0.5330	0.4630	0.4081
	200	0.6797	0.6295	0.5789	0.5289	0.4599	0.4055
	300	0.6724	0.6239	0.5742	0.5252	0.4567	0.4029
	400	0.6685	0.6210	0.5718	0.5233	0.4550	0.4016
Compression factor *a*_*v*_/MPa^–1^	50	0.3349	0.2572	0.2164	0.1859	0.1595	0.1392
	100	0.2404	0.1991	0.1587	0.1394	0.1196	0.0994
	200	0.0902	0.0689	0.0529	0.0403	0.0318	0.0263
	300	0.0730	0.0556	0.0465	0.0372	0.0318	0.0263
	400	0.0386	0.0290	0.0240	0.0194	0.0170	0.0128
Compression modulus *Es*/MPa	50	5.0782	6.4016	7.3574	8.2833	9.2093	10.1541
	100	7.0232	8.2188	9.9828	10.9944	12.2290	14.1657
	200	18.6286	23.6651	29.8485	37.9577	45.9721	53.5000
	300	22.9118	29.2164	33.8655	41.0208	45.8721	53.4000
	400	43.1778	55.8286	65.3733	78.6600	85.6609	109.6667

#### Effects of content on void ratio

**Figure 2:**
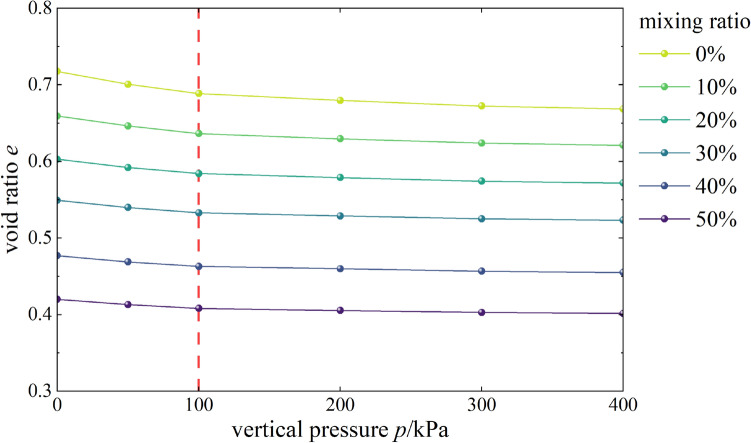
*e-p* correlation curves.

The void ratio-vertical pressure curves of mixed soil samples of loess and burnt rock particles are shown in Fig. 2. As observed, the void ratio of samples with different contents of burnt rock-solid waste particles gradually decreased with the increase of vertical pressure. When the vertical pressure was lower than 100 kPa, the curve was relatively steep and the gradient was large, suggesting that samples with different contents of burnt rock-solid waste particles decreased significantly; When the vertical pressure was more significant than 100 kPa, the curve gradually slowed down, the decreasing trend of the void ratio also gradually weakened and the gradient was small, suggesting that mixed soil samples with different contents of burnt rock-solid waste particles had higher compressibility under low-pressure conditions. In contrast, their compressibility gradually decreased with increased vertical pressure.

**Figure 3:**
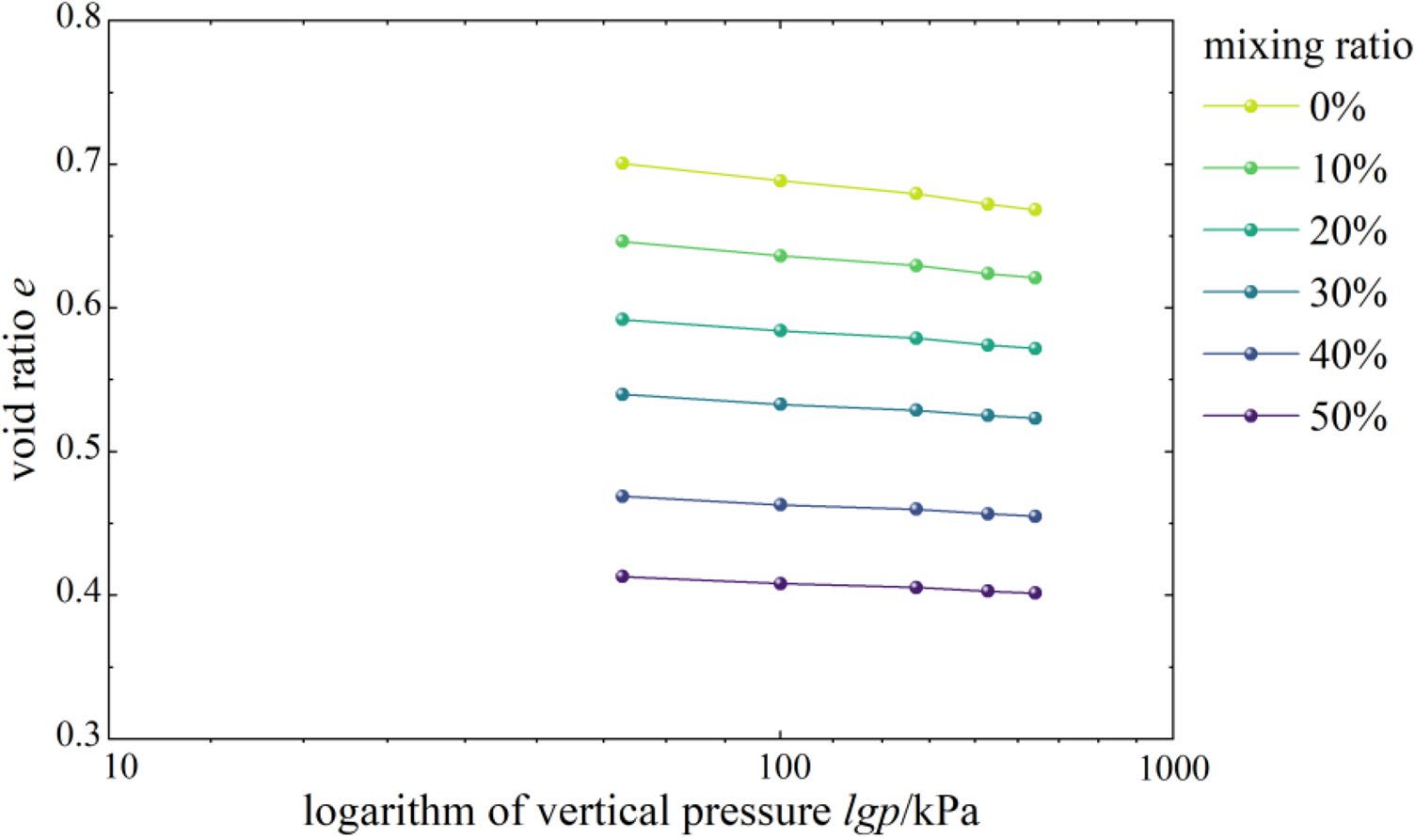
*e-*lg*p* correlation curves.

According to Fig. 3, the *e-*log*p* curves of mixed soil samples with 0 ~ 50% burnt rock particles were all approximately linear, and the linear gradient was very close. The specific difference was that in the case of low strength load (< 100 kPa), the *e-*log*p* curves of mixed soil samples with different contents of burnt rock-solid waste particles were relatively steeper, and the gradient was more significant, suggesting that the void ratio of samples with different contents of burnt rock-solid waste particles had significantly reduced; however, when the vertical pressure was more significant than 100 kPa, the curve gradually slowed down, and the decreasing trend of the void ratio also gradually weakened and finally tended to be stable.

#### Effects of content on the coefficient of compressibility

**Figure 4:**
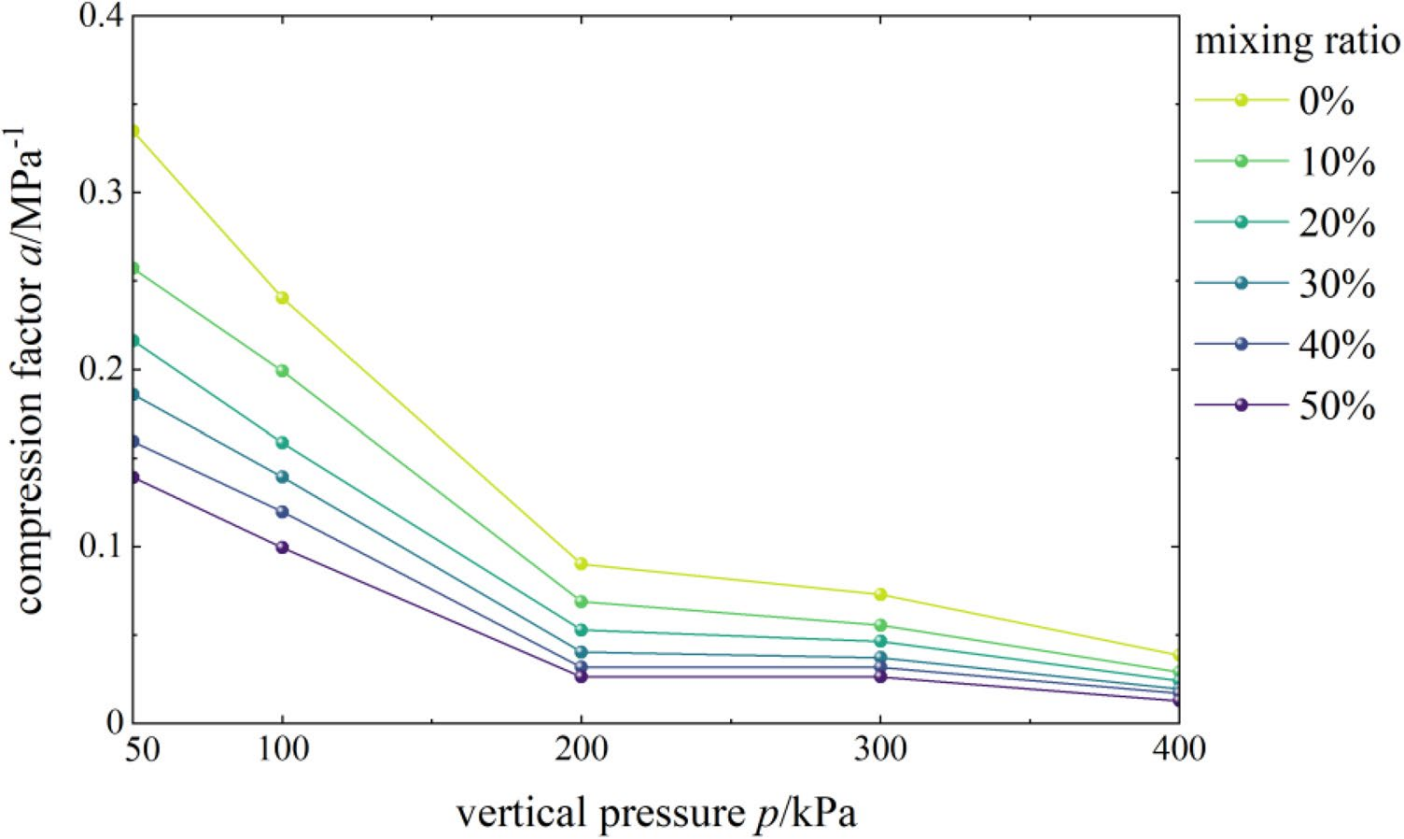
*a-p* correlation curves.

Figure 4 shows the curves of the compressibility coefficient and vertical pressure of mixed soil samples of loess and burnt rock particles. As seen, the coefficient of compressibility a of mixed soil samples of loess and burnt rock particles decreased with the increase of vertical pressure. Typically, the coefficient of compressibility (denoted as a1-2) corresponding to the vertical pressure of 100 kPa and 200 kPa was used to evaluate the soil compressibility. The test showed that the a1-2 of pure compacted loess, 10%, 20%, 30%, 40%, and 50% mixed soil were 0.0902 MPa^–1^, 0.0689 MPa^–1^, 0.0529 MPa^–1^, 0.0403 MPa^–1^, 0.0318 MPa^–1^ and 0.0263 MPa^–1^ respectively. Therefore, mixed soil samples with different contents of burnt rock-solid waste particles all exhibited low compressibility (a1–2 < 0.1 MPa^–1^). Additionally, as the burnt rock particle content increased, their compressibility coefficient a gradually decreased. The results showed that increasing the burnt rock particle content effectively reduces the compressibility coefficient of mixed soil and thus reduces the building settlement.

**Figure 5:**
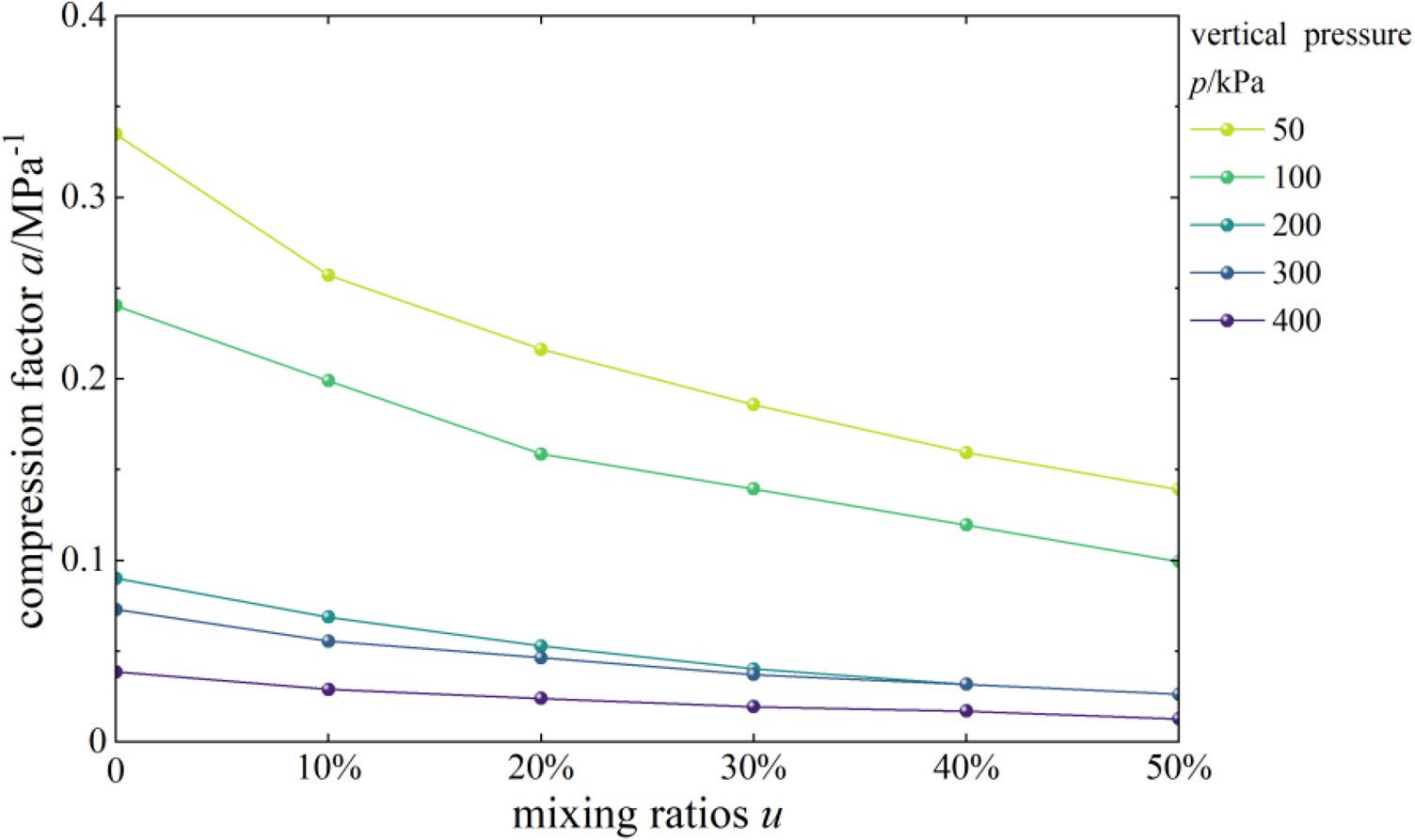
*a-u* correlation curves.

Figure 5 shows the curves of the coefficient of compressibility of mixed soil samples as a function of the content of burnt rock particles. As observed, the coefficient of compressibility a of mixed soil samples of loess and burnt rock particles showed a decreasing trend with the increase of burnt rock particle content, suggesting that as the content of burnt rock particles increased, the coefficient of compressibility a of mixed soil samples of loess and burnt rock particles decreases gradually. The coefficient of compressibility a of the mixed soil sample with 50% burnt rock-solid waste particles was the smallest, and the compaction effect of the mixed soil was also the best at this time. However, when the vertical pressure was lower than 100 kPa, the curve was relatively steep, and the gradient was significant, indicating that the coefficient of compressibility a of samples with different contents of burnt rock-solid waste particles decreased significantly; when the vertical pressure was significant than 100 kPa, the curve gradually slowed down, the decreasing trend of the coefficient of compressibility *a* also gradually weakens, and the gradient was slight. The reasons for the above phenomenon were: mixed soil samples with different contents of burnt rock-solid waste particles have higher compressibility under low-pressure conditions, while its compressibility gradually decreased with the gradual increase of vertical pressure; that is, the compressibility of the sample was higher under low pressure.

#### Effects of content on compressive modulus

**Figure 6:**
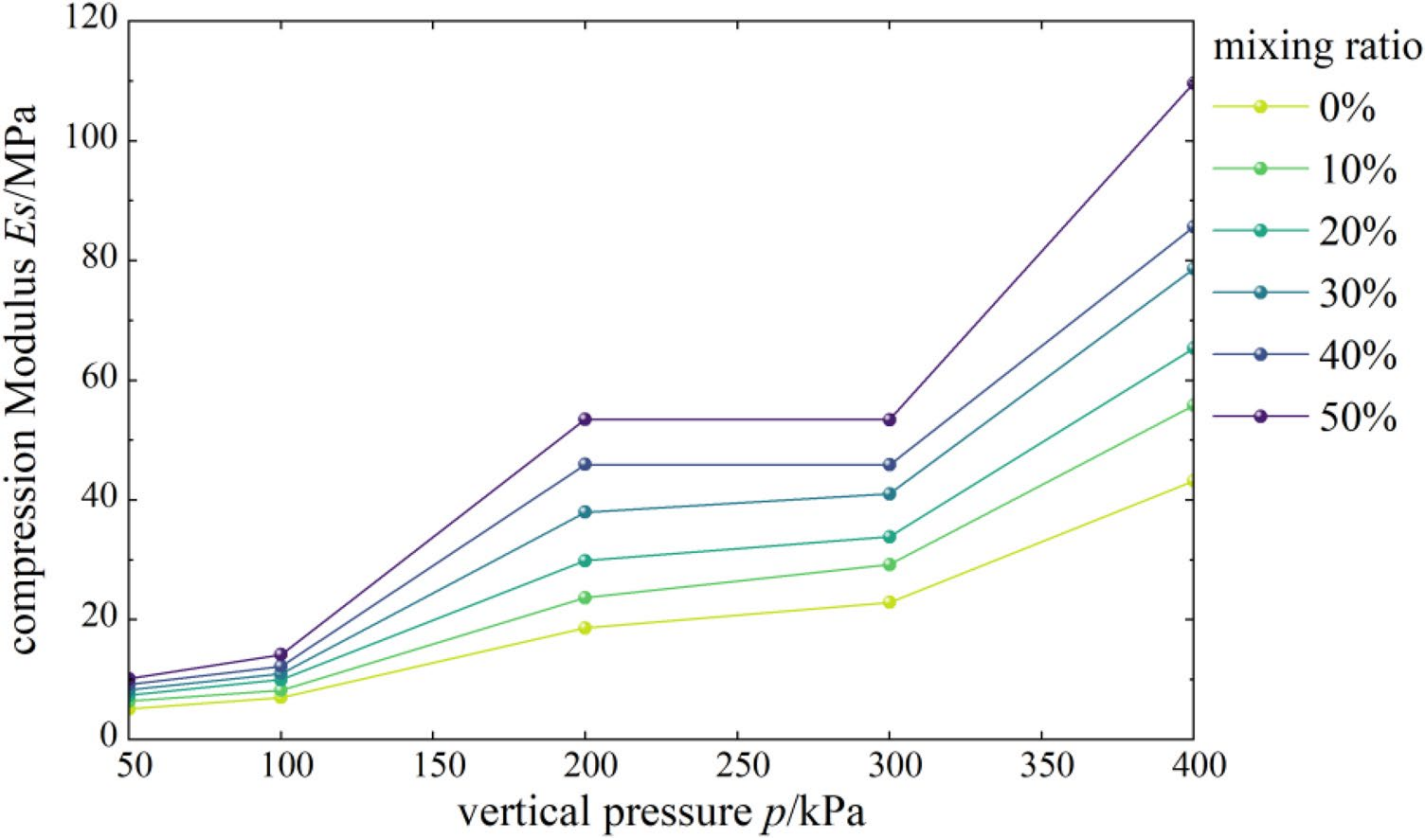
*Es-p* correlation curves.

Figure 6 shows the curves of compressive modulus vs. vertical pressure of mixed soil samples of loess and burnt rock particles. As shown, the compressive modulus of the composite soil samples with different contents of burnt rock-solid waste particles increased first, then decreased, and finally increased with vertical pressure. At the same time, the overall trend showed an increase. When the vertical pressure was lower than 100 kPa, the compressive modulus of mixed soil was similar to that of pure loess. However, when the vertical pressure was higher than 100 kPa, the compressive modulus of mixed soil was significantly higher than that of pure loess.

**Figure 7:**
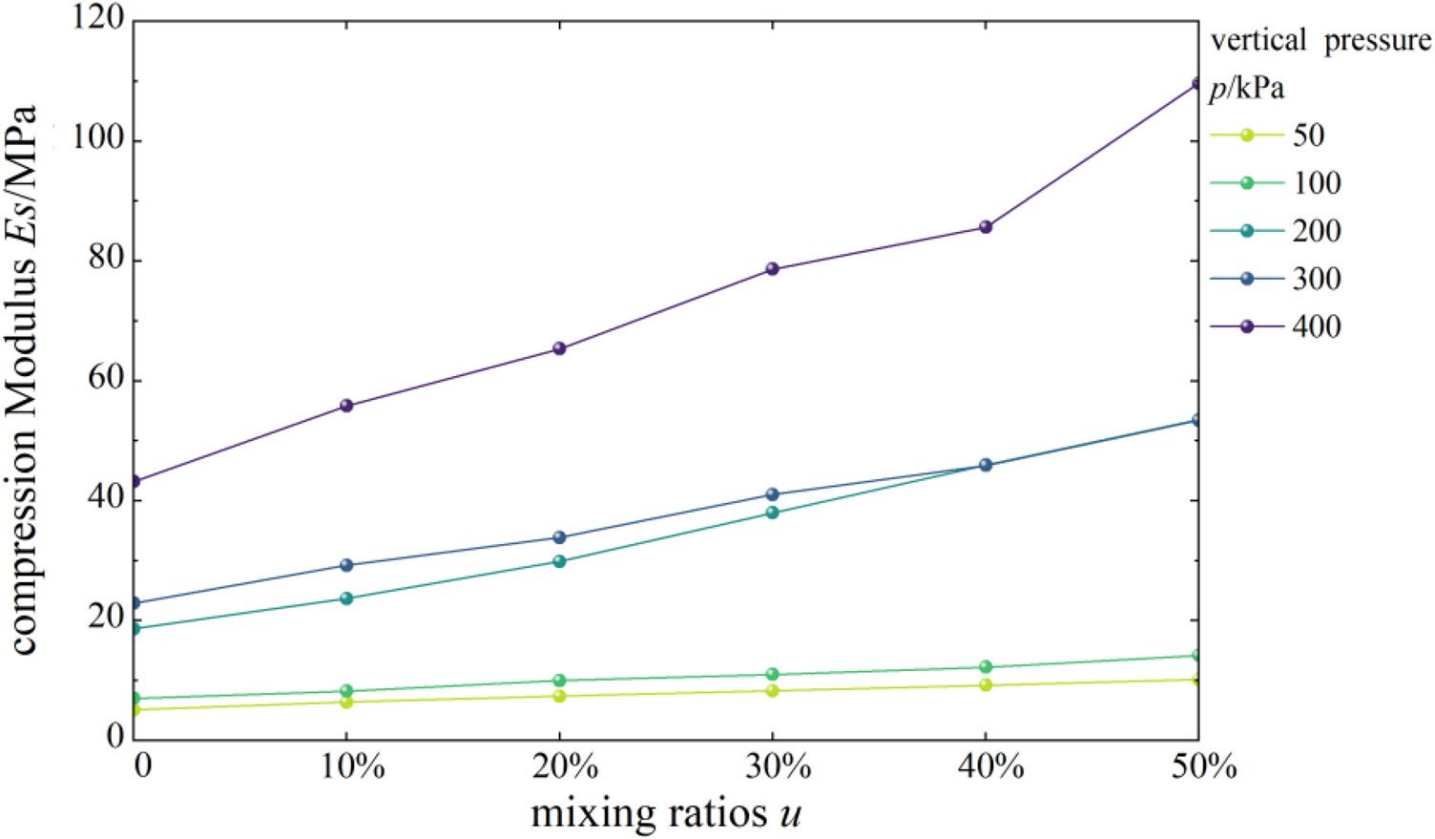
*Es-u* correlation curves.

Figure 7 shows the compressive moduli of mixed soil samples of loess and burnt rock particles as a function of the content of burnt rock particles. As seen, the compressive modulus of the composite soil sample showed a gradual increase with increased burnt rock particles’ content. Among them, when the range of burnt rock particles was 50%, the compressive modulus of mixed soil was the largest. That is, the bearing capacity of 50% mixed soil was the highest, the compressibility was the lowest, and the compaction effect was the best.

### Modified loess strength parameter: shear strength test

The shear stress of the mixed soil sample with different contents of burnt rock particles under varying levels of vertical pressure is shown in Table 6. The curves fit according to the relationship between shear stress and vertical pressure. The shear strength of mixed soil samples with different contents of burnt rock-solid waste particles was obtained (Table 7).

**Table 6:** Shearing stress for different mixing ratios.

Parameter	Vertical pressure *p*/kPa	0%	10%	20%	30%	40%	50%
Shearing stress *τ*/kPa	100	9.6	12	15.6	18	20.16	24
	200	18	26.4	30	32.4	36	45.6
	300	28.8	37.2	42	46.8	56.4	62.4
	400	36	46.2	50.4	55.2	60	73.2

**Table 7:** Shearing strength for different mixing ratios.

Mixing ratio *u*	0%	10%	20%	30%	40%	50%
Cohesion *c*/kPa	0.6	2.1	5.4	6.6	8.16	10.2
Internal friction angle *φ*/°	5.14	6.47	6.64	7.18	7.96	9.34

#### Effects of content on cohesion

**Figure 8:**
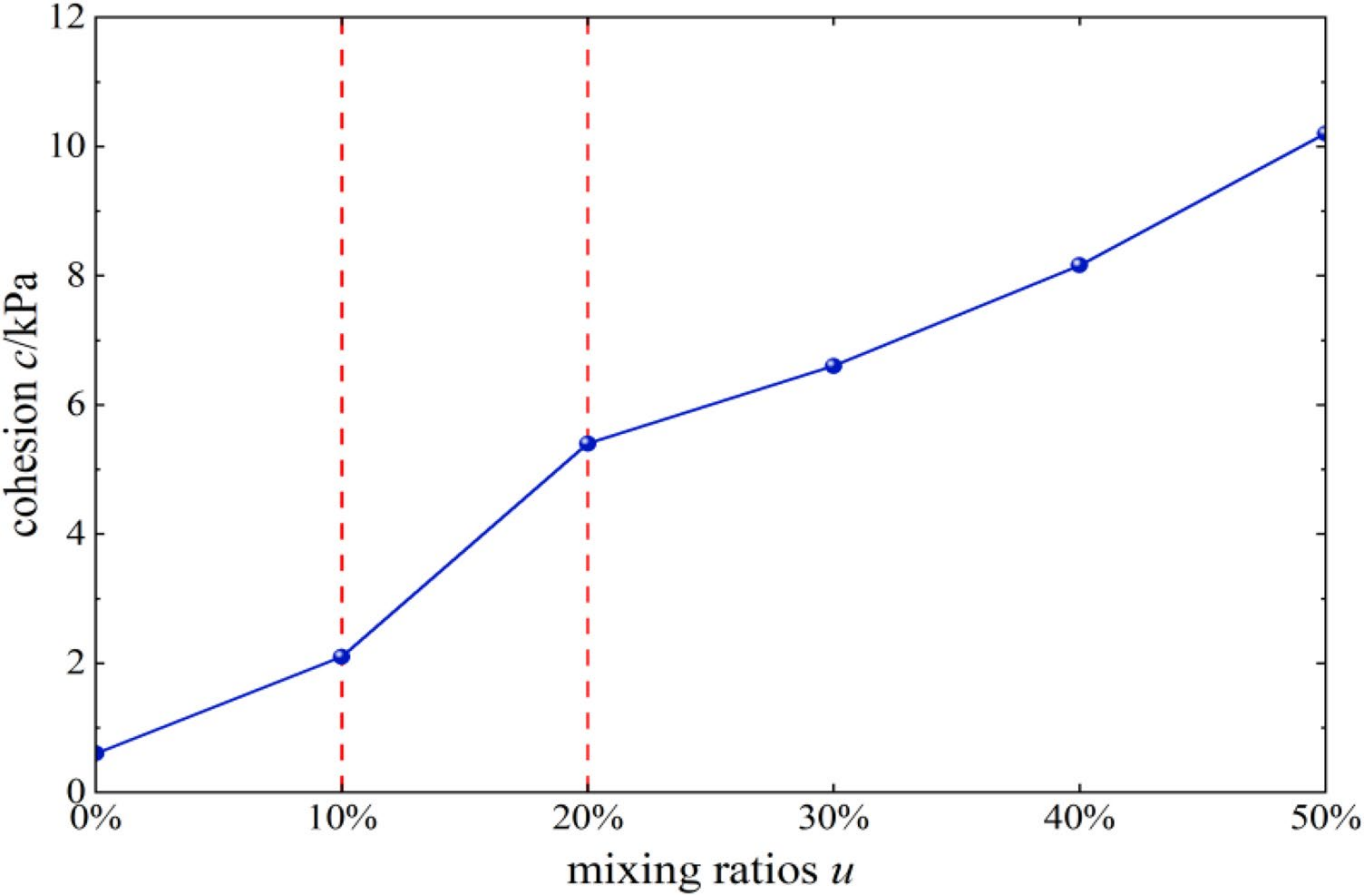
*c-u* correlation curves.

Figure 8 shows the trends of cohesion c of mixed soil samples of loess and burnt rock particles as a function of the content of burnt rock particles. As observed, the cohesion of each diverse soil sample generally showed an increasing trend with increased burnt rock particle content. When the range of burnt rock particles was 10–20%, the linear gradient changed the most, indicating that the cohesive force of mixed soil samples of loess and burnt rock particles had the most noticeable improvement in this range.

#### Effects of content on the angle of internal friction

The angle of internal friction *φ* of mixed soil samples of loess and burnt rock particles increased gradually as the content of burnt rock particles increased. When the content of burnt rock particles was 10–20%, the linear gradient changed the least, indicating that the angle of internal friction of mixed soil samples of loess and burnt rock particles did not change much in this range, and the improvement effect was the least obvious. At the same time, it was in sharp contrast with the influence of content on the cohesion in this range, indicating that the strength of burnt rock particles-modified loess shear was mainly achieved by increasing cohesion.

#### Effects of content on shear strength

**Figure 9:**
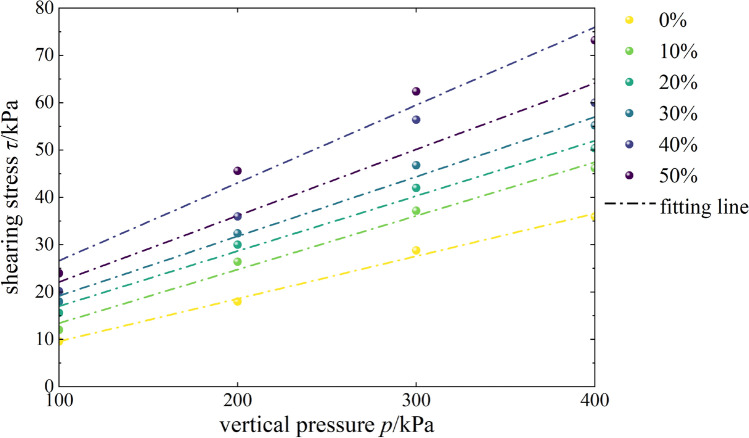
*τ-p* correlation curves.

As shown in Fig. 9, the shear stress of samples with different contents of burnt rock-solid waste particles increased gradually with the increase of vertical pressure, indicating that as the content of burnt rock particles increased, the shear strength indexes of mixed soil samples of loess and burnt rock particles increased gradually, demonstrating that burnt rock particles significantly improved the shear strength of loess. According to curves of samples with different contents of burnt rock-solid waste particles, the higher the content of burnt rock particles, the larger the gradient and intercept of the Coulomb straight line, indicating that the shear strength was also better. And when the content of burnt rock particles reached 50%, the shear strength of mixed soil samples of loess and burnt rock particles was the largest, and the shear resistance of mixed soil was also the best at this time.

According to Table 8, the fitted mathematical expressions of samples with different contents of burnt rock particles were linearly and positively correlated (correlation coefficient *R*^*2*^ = 0.94–0.99), indicating high goodness of fit; This was also in line with Coulomb’s law of shear strength, which showed that there was a linear relationship between the shear strength of mixed soil samples of loess and burnt rock particles and the normal stress on the shear plane at the general stress level.

**Table 8:** Fitting formulas for different mixing ratios.

Number	Mixing ratio%	Fitting formula	*R*^*2*^
1	0	*τ* = 0.09*p* + 0.6	0.99
2	10	*τ* = 0.1134*p* + 2.1	0.99
3	20	*τ* = 0.1164*p* + 5.4	0.99
4	30	*τ* = 0.126*p* + 6.6	0.99
5	40	*τ* = 0.1399*p* + 8.16	0.94
6	50	*τ* = 0.1644*p* + 10.2	0.98

## Discussion

### Qualitative analysis of micro-structure of modified loess

**Figure 10:**
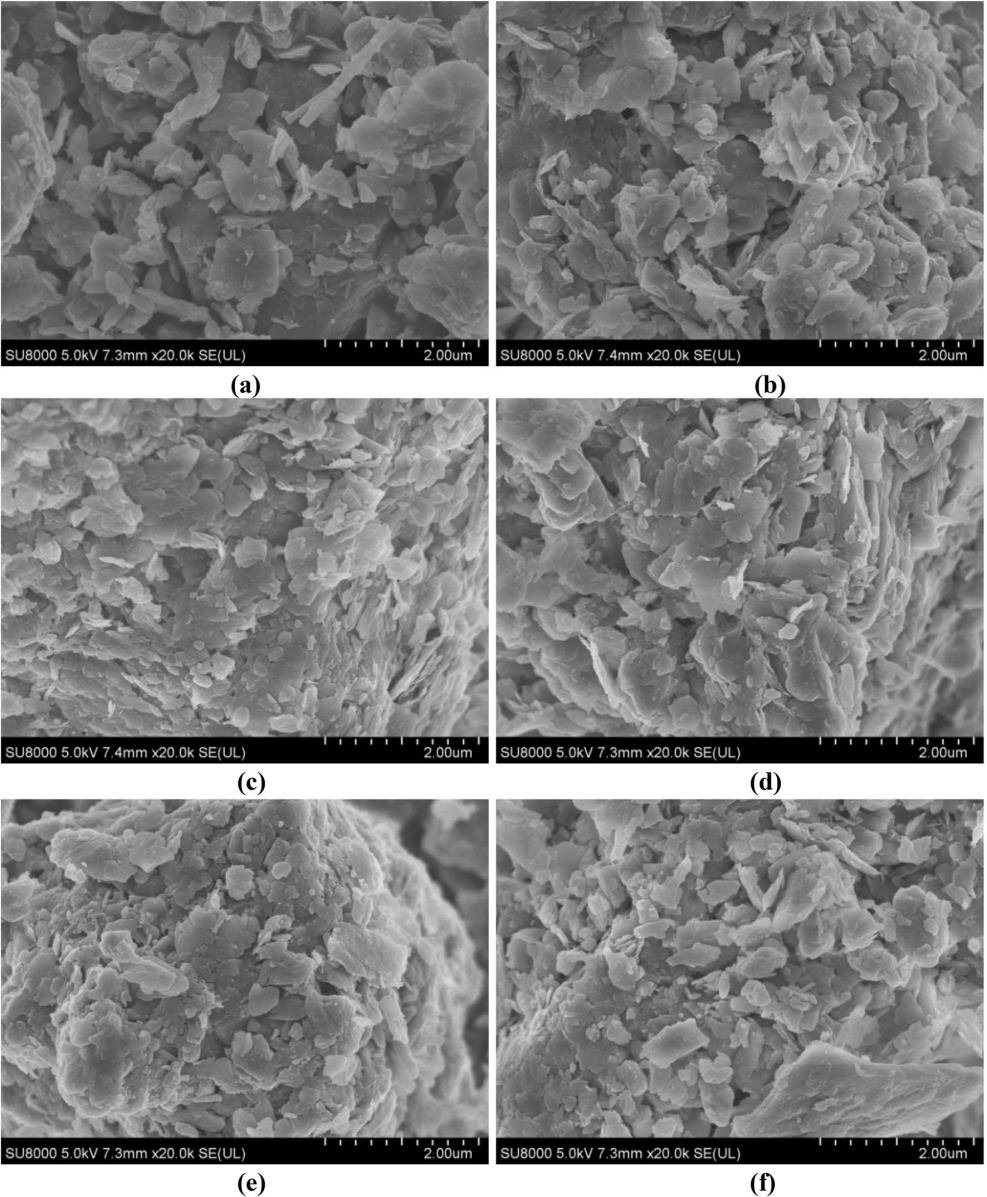
Microstructure at 20,000 times magnification with different mixing ratio. (**a**) Pure loess; (**b**) Mixed with 10% burnt rock particles; (**c**) Mixed with 20% burnt rock particles; (**d**) Mixed with 30% burnt rock particles; (**e**) Mixed with 40% burnt rock particles; (**f**) Mixed with 50% burnt rock particles.

To compare and analyze the micro-structure characteristics of pure loess and modified loess, loess samples with 0, 10%, 20%, 30%, 40%, and 50% of burnt rock particles were prepared and tested, and performed the qualitative analysis at 20,000 times. It can be seen from Fig. 10a that when the content of burnt rock particles is 0, the skeleton particles of loess were mainly single grains, the shape of the particles was irregular, the arrangement was loose, the pores between the loess particles were large and the contact area smaller.

#### Micro-mechanism analysis of modified loess deformation

As seen from Fig. 10b–f that when burnt rock particles were added, the skeletal particles of the mixed soil were mainly agglomerated, the shape of the particles was irregular, the arrangement was relatively tight, and the pores between the mixed soil particles were reduced; improving the particle size distribution of loess. Since the particle size of the burnt rock was more significant than that of loess, incorporating burnt rock particles and increasing burnt rock particles’ content gradually became the skeleton structure of the mixed soil. A small amount of loess fills the pores of burnt rock particles; it can be seen that the pores are gradually decreasing. Meanwhile, as the content of burnt rock particles continued to increase, the pores between its mixed soil particles were filled and cemented. Furthermore, the particles were inter-connected to form larger aggregates. As a result, the inter-particle pores were filled to varying degrees, making the modified soil structure smaller and smaller than the plain soil pores, improving the compressibility of the loess, and effectively solving the uneven building settlement.

#### Micro-mechanism analysis of modified loess strength

It is seen from Fig. 10b–f that the micro-structure of mixed soil increased with the content of burnt rock particles, the contact area between particles became more extensive, specifically including between burnt rock particles, between loess particles, the contact area between burnt rock particles and loess particles, which makes the frictional force of inter-particle interaction more extensive, improving the shear strength of mixed soil samples of loess and burnt rock particles. Additionally, as the content of burnt rock particles increased, there was a position change between burnt rock particles and loess particles when subjected to shear stress, making the loess particles densely surround the burnt rock particles, forming an agglomeration. Consequently, the pores between the particles were further reduced. Moreover, the agglomerated structure gradually strengthened the “occlusion action” between particles, increasing the cohesion c of the modified loess. Meanwhile, due to the irregular shape of the burnt rock particles and the rough surface, the angle of internal friction ψ of the modified loess gradually increased.

### Quantitative analysis of modified loess micro-structure

**Figure 11:**
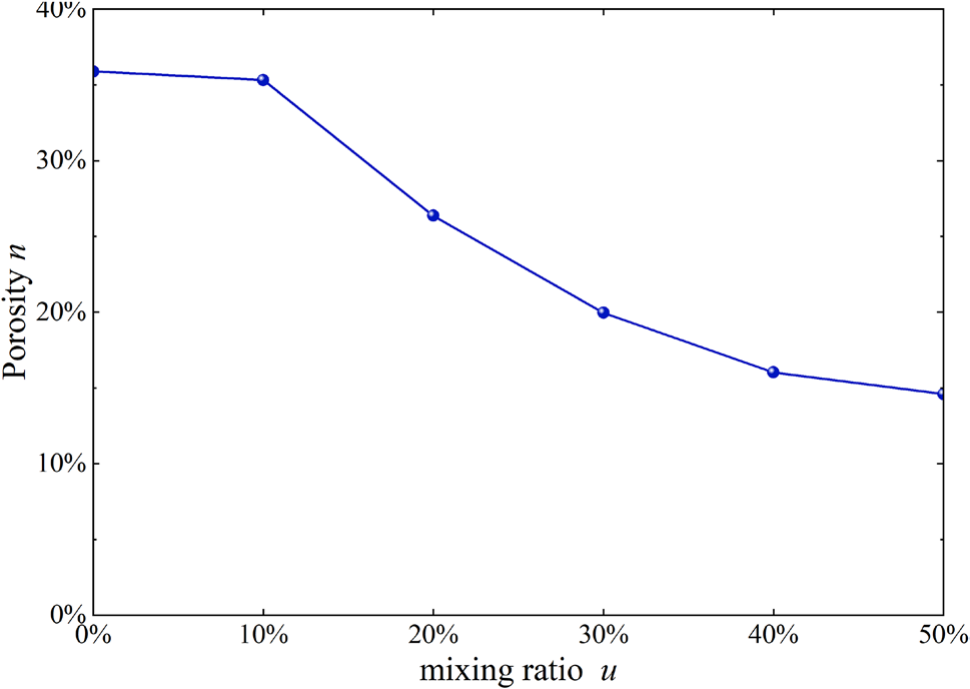
*n-u* correlation curves.

**Figure 12:**
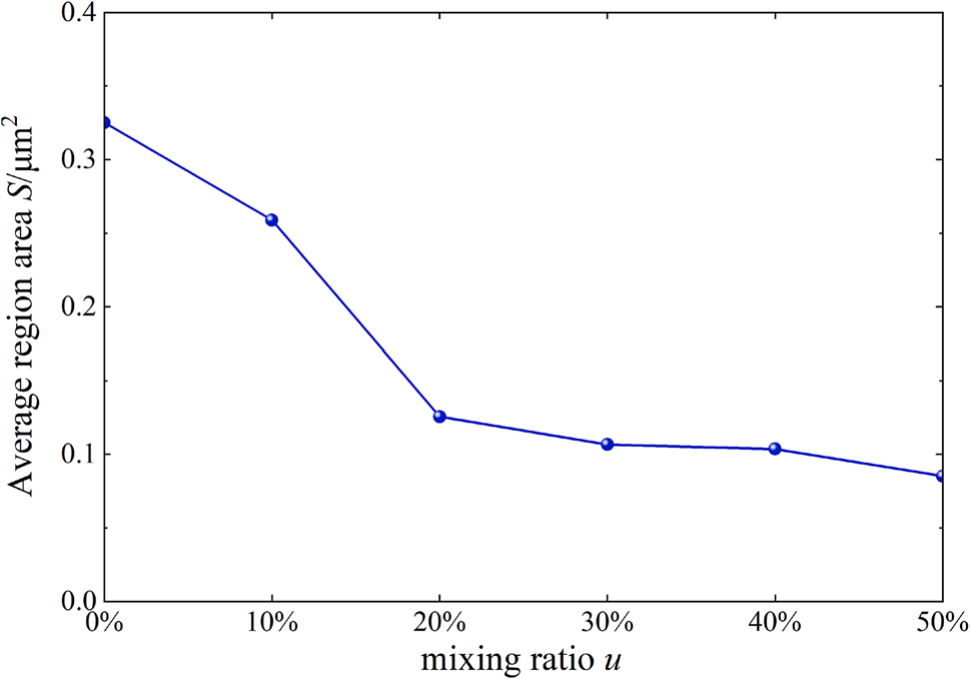
*S-u* correlation curves.

To further characterize the parameters related to the micro-structure, the particle (pore) and crack image recognition and analysis system (PCAS) was used for microscopic quantitative analysis of SEM images, and the parameters such as porosity, average area, and content curves were obtained^30^. Porosity, average area, and content curves are shown in Figs. 11 and 12.

**Figure 13:**
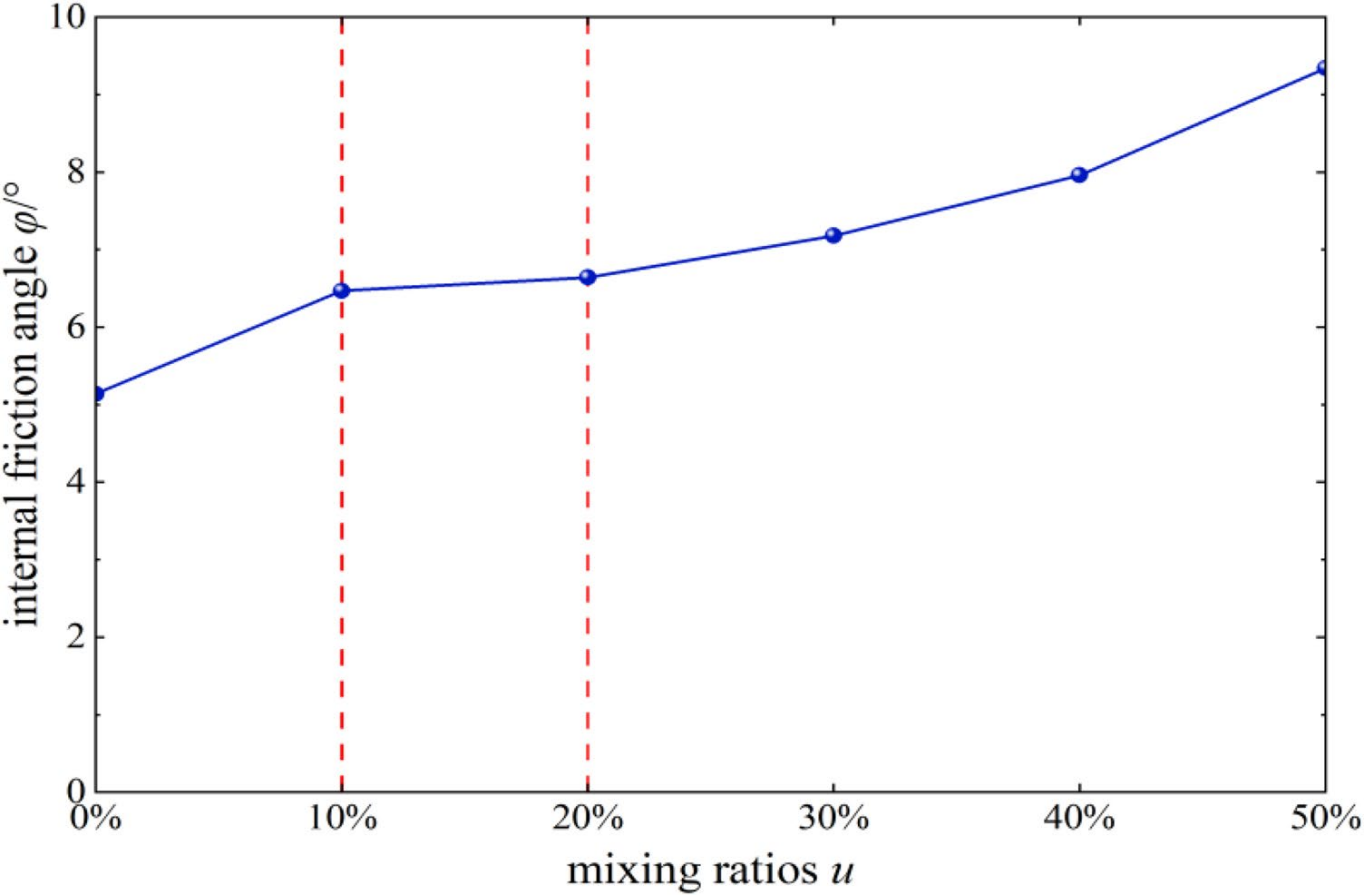
*φ-u* correlation curves.

As shown in Figs. 11 and 12, mixed soil’s porosity and average particle size showed a downward trend with increased burnt rock particle content. Among them, the porosity decreased from 35.9 to 15.1%, and the average area decreased from 0.33 to 0.09 mm^2^, suggesting that modification by burnt rock-modified loess improved compressive deformation and shear strength of engineering soil, which were characterized by soil porosity. The soil porosity and average area were reduced through burnt rock-modified loess, improving soil compressive deformation and shear strength, which was beneficial for engineering construction and disaster prevention and mitigation in loess areas. Additionally, when the content of burnt rock particles was 10–20%, the linear gradient changed the most, which was consistent with the experimental results of Figs. 8 and 13 that the soil’s shear strength improvement was most significant within the content range.

## Conclusions

The deformation damage properties of modified loess were investigated by compression-consolidation test and shear strength test, and the microscopic mechanism of modified loess was clarified using SEM. The main conclusions of this article are as follows:

1. As the content of burnt rock-solid waste particles increased, the void ratio and coefficient of compressibility of all mixed soil samples decreased as the vertical pressure increased, while the compressive modulus first increased and then decreased and then increased with the increase of vertical pressure (overall increase).
2. The cohesion and angle of internal friction ψ of the mixed soil samples showed an overall increasing trend with the increase of the content of burnt rock-solid waste particles. Furthermore, as the pores in the modified loess were filled and cemented with each other, the contact area increased, the “occlusion action” between the particles gradually strengthened, and the shape of the burnt rock-solid waste particles became irregular, significantly improving the compressibility, cohesive force and angle of internal friction of the modified loess.
3. By using the burnt rock-solid waste particles modified material with stable performance, the bearing capacity and shear strength of modified loess were significantly improved by changing the contact situation of the internal particles of the loess and reducing the inner pores of the soil, thereby increasing the strength of foundation soil.

## Data Availability

The datasets analysed during the current study available from the corresponding author on reasonable request.
